# ﻿Revision of the Afrotropical genus *Protoleptops* Heinrich, 1967 (Hymenoptera, Ichneumonidae, Ichneumoninae), with description of a new species from Burundi

**DOI:** 10.3897/zookeys.1214.131071

**Published:** 2024-10-07

**Authors:** Davide Dal Pos, Augustijn De Ketelaere, Filippo Di Giovanni

**Affiliations:** 1 Department of Biology, University of Central Florida, Orlando, FL 32816, USA University of Central Florida Orlando United States of America; 2 Beernem, Belgium Unaffiliated Beernem Belgium; 3 Department of Life Sciences, University of Siena, Siena, Italy University of Siena Siena Italy

**Keywords:** **Key words**: Biodiversity, Darwin wasps, identification key, new records, parasitoids, taxonomy

## Abstract

This study presents a comprehensive revision of the genus *Protoleptops* Heinrich, 1967. We describe a new species, *P.nyeupe* Dal Pos & Di Giovanni, **sp. nov.**, from Burundi, marking the first documented occurrence of an Ichneumoninae species in the country. Additionally, we provide the first diagnostic description of the female *P.farquharsoni* Heinrich, 1967 and report a new occurrence of this species in KwaZulu-Natal. Furthermore, we document *P.magnificus* for Mpumalanga (South Africa) and *P.angolae* Heinrich, 1967 in Uganda, thereby extending the known range of the latter into East Africa. A detailed catalogue of all species within the genus *Protoleptops* is also included.

## ﻿Introduction

Among the extant subfamilies of Darwin wasps, Ichneumoninae stands out as the most diverse, with over 4300 species in 430 genera (Yu et al. 2016; Santos et al. 2021). After a troubled past, marked by several inconsistent and paraphyletic tribal subdivisions, the subfamily Ichneumoninae is currently divided into seven monophyletic tribes, with Ichneumonini comprising the vast majority of the taxa (Santos et al. 2021). In the Afrotropics, Heinrich (1967) recognized 12 tribes of Ichneumoninae (and did not deal with Phaeogenini), of which nine have been synonymized under Ichneumonini (Santos et al. 2021). Among these dissolved tribes, Protichneumonini was recognized by having: (1) the area dentipara gradually curving down on the propodeum, almost reaching the hind coxa; (2) frons smooth, without longitudinal carina; (3) propodeum not abbreviated into a boss or arch; (4) head not cubical; (5) face and clypeus with a distinct epistomal sulcus (also called clypeal suture); and (6) mandibles not or only slightly twisted (e.g., Heinrich 1961; Heinrich 1967).

Within Protichneumonini, Heinrich (1967) described two new genera: the monotypic *Protoleptops* Heinrich, 1967, comprising only *Protoleptopsheinrichi* Heinrich, 1967, and *Apatetorops* Heinrich, which contained three new species – *Apatetoropsangolae* Heinrich, 1967, *A.farquharsoni* Heinrich, 1967, and *A.magnificus* Heinrich, 1967. Heinrich (1967) proposed mainly the following characters for the separation of the two genera: the carination of the propodeum (well developed in *Protoleptops*, extremely reduced in *Apatetorops*) and the constriction of T2–T5 (constricted in *Protoleptops*). Later on, Townes and Townes (1973) recognized *Apatetorops* as a junior synonym of *Protoleptops*, without providing any reasoning for their action but probably not considering the above characters as sufficient for the separation of the two genera. Since then, the genus has never been reviewed nor recorded.

In the current contribution, we provide a review of *Protoleptops*, with an updated diagnosis of the genus, the first diagnosis of the female of *P.farquharsoni*, and the description of a new species, *P.nyeupe* Dal Pos & Di Giovanni, sp. nov. from Burundi. Additionally, a commented catalogue of the species is presented, together with some additional new distributional records.

## ﻿Materials and methods

### ﻿Photographs

An OPTIKA SZM-2 dissecting stereo microscope was used for observation and study. Photographs of *Protoleptopsnyeupe* Dal Pos & Di Giovanni, sp. nov. were taken with a Canon EOS 7D, Canon MP-E 65 mm f/2.8 1–5 × macro lens and a Canon EF 100 mm macro lens. Zerene Stacker software ver. 1.04 was used for the stacking. Images were enhanced using Adobe Photoshop ver. 24.4.1. All the other pictures were taken using an Olympus OM-D camera mounted on a Leica M125 C binocular microscope and stacked using Helicon Focus (ver. 8).

### ﻿Mapping

Maps were generated in QGIS ver. 3.2 using the ESRI Imagery plugins (https://www.esri.com), integrated into the Python console for QGIS for the main background layer, and an overlaid globe projection using the Thematic Mapping in the Thematic Mapping Engine integration. Following Dal Pos et al. (2023), the distribution for Madagascar follows the official division, which recognizes 23 regions (“faritra”) instead of the former six provinces (INSTAT 2010).

### ﻿List of depositories

**DDPC** Davide Dal Pos private collection, Orlando, Florida, USA;

**FSCA**Florida State Collection of Arthropods, Gainesville, Florida, USA (Elijah Talamas);

**MNHN**Muséum national d’Histoire naturelle**MZUR** Museum of Entomology, “Sapienza” University of Rome, Italy (Maurizio Mei);

**OÖLM** Oberösterreichische Landesmuseen, Linz, Austria;

**ZSM**Zoologische Staatssammlung München, Munich, Germany (Olga Schmidt).

### ﻿Data of examined material

Label information for the type specimens is reported verbatim, using the following conventions: / = different lines; // = different labels; italic = handwriting. For non-type specimens, names of collecting localities have been standardized.

### ﻿Treatment of taxa

Morphological terminology follows Broad et al. (2018) and is aligned with the Hymenoptera Anatomy Ontology (Yoder et al. 2010). However, unlike Broad et al. (2018), we used the following terms: “mesoscutellum” instead of “scutellum” [see Dal Pos et al. (2024) for more details]; and “epistomal sulcus” instead of “clypeal sulcus”. We also decided to employ “propodeum” instead of the HAO-suggested term “metapectal-propodeal complex” simply because the former is more widely used. For propodeal carinae and areas, we adhered to the terminology used by Broad et al. (2018) and we did not align it with the HAO, as this terminology is almost completely absent from the database. In addition, we reported the widely used term “costula” to indicate the portion of the anterior transverse carina dividing the area externa from the area dentipara. Names of metasomal tergites are abbreviated as T1 (first metasomal tergite), T2 (second metasomal tergite), etc.

For each species, a differential diagnosis, type information, material examined, and relevant comments are provided. Type localities are reported as they appeared in the original publication with the addition of the country of origin. Unavailable names are identified in square brackets (as in Dal Pos et al. 2023).

## ﻿Results

### ﻿Key to the species of *Protoleptops* Heinrich, 1967

**Table:** 

1	Carination of propodeum nearly complete (Fig. 4D, 6B): lateral portion of anterior transverse carina (i.e., costula) strong, also lateral longitudinal carina at the level of area dentipara distinct, even forming a slight ridge between area dentipara and area spiracularis (Fig. 6B). Scopa absent (Fig. 4C). T2–T5 each anteriorly constricted (Fig. 4E)	***P.heinrichi* Heinrich, 1967**
–	Carination of propodeum incomplete (Figs 1D, 3B, 5D, 6E, 7B): anterior transverse carina lacking or at least incomplete (i.e., costula absent or only hinted), area dentipara confluent with area externa and area spiracularis. Scopa present (Figs 1E, 2C, 5E, 7E). T2–T5 not anteriorly constricted (Figs 1F, 2E, 3B, 3E, 5F, 6E, 7B)	**2**
2	Temples straight (not bulging) in dorsal view (Figs 1C, 7C). Hind tarsus white marked	**3**
–	Temples strongly bulging in dorsal view (Figs 2D, 5C). Hind tarsus brownish-black	**4**
3	T2 medially densely punctate (Fig. 1F, 3B). In females, mesoscutellum reddish-orange, mesoscutum black laterally, reddish-orange medially (Fig. 1D). Scopa small, reduced to a tuft of setae on ventro-distal area of the hind coxa (Fig. 1E). T2 black with a posterior white band (Fig. 1F, 3B). Area petiolaris not delimited, confluent with area basalis and area superomedia (Fig. 1D, 3B). Mesopleuron densely and strongly punctate (Fig. 1A, 3A)	***P.angolae* (Heinrich, 1967)**
–	T2 medially strongly longitudinally striate (Fig. 7B). In females, mesoscutellum and mesoscutum completely white (Fig. 7A). Scopa mid-sized, covering 1/3 of the ventro-distal area of the hind coxa (Fig. 7E). T2 completely reddish-brown, without posterior white band (Fig. 7B). Area petiolaris separated from area superomedia (Fig. 7B). Mesopleuron superficially and sparsely punctate	***P.nyeupe* Dal Pos & Di Giovanni, sp. nov.**
4	T2 strongly longitudinally striate; T3–T8 smooth and shining (Fig. 5F, 6E). Mesoscutum sparsely and superficially punctate throughout; mesoscutellum with lateral white bands, reddish-brown medially (Fig. 5C). Scopa large, covering roughly 2/3 of the ventro-distal area of the hind coxa (Fig. 5E)	***P.magnificus* (Heinrich, 1967)**
–	T2 medially densely punctate; T3–T8 shagreened (Fig. 2E, 3E). Mesoscutum densely punctate anteriorly; mesoscutellum completely reddish-orange (Fig. 2D). Scopa reduced to a tuft of setae on ventro-distal area of the hind coxa (Fig. 2C)	***P.farquharsoni* (Heinrich, 1967)**

### ﻿Taxonomy

**Class Insecta Linnaeus, 1758**

**Order Hymenoptera Linnaeus, 1758**

**Superfamily Ichneumonoidea Latreille, 1802**

**Family Ichneumonidae Latreille, 1802**

**Subfamily Ichneumoninae Latreille, 1802**

**Tribe Ichneumonini Latreille, 1802**

#### ﻿Genus Protoleptops

Taxon classificationAnimaliaHymenopteraIchneumonidae

Heinrich, 1967

F24978BD-E31B-5EF9-9F4D-6C6C5FAEF302

Protoleptops Heinrich, 1967: 71–72. Type species Protoleptopsheinrichi Heinrich, 1967, by original designation.
Apatetorops Heinrich, 1967: 79–81. Type species Apatetoropsmagnificus Heinrich, 1967, by original designation. Synonymized by Townes and Townes (1973: 226).

##### Diagnosis.

We hereby provide a brief diagnosis of *Protoleptops* by including the traits of its junior synonym *Apatetorops*, therefore expanding the concept of the genus. We discovered a new character that separates well the former two genera that was not reported by Heinrich (1967): the presence/absence of a scopa on the ventral section of the hind coxa. The dimension of the scopa also works well in separating some of the species (see key above). A phylogenetic analysis with the inclusion of more specimens will be necessary to understand if the synonymy of the two genera proposed by Townes and Townes (1973) still stands. Since *Apatetorops* and *Protoleptops* were included by Heinrich (1967) in the now-dissolved tribe Protichneumonini, we invite the reader to either use Heinrich’s (1967) key to the Afrotropical tribe or review our diagnosis in the Introduction. Within this former tribe, *Protoleptops* can easily be distinguished from all the other Afrotropical genera of Ichneumoninae by the following combination of characters: (1) carination of the propodeum not fully complete, at least with area superomedia and area basalis confluent (divided in *Chasmopygium* Heinrich, 1967 and *Holcichneumon* Cameron, 1911); (2) postpetiole with either irregular striation or puncto-striate (uniformly and densely punctate in *Aethiamblys* Heinrich, 1967, *Afrocoelichneumon* Heinrich, 1938, *Corymbichneumon* Morley, 1919, and *Punctileptops* Heinrich, 1967); (3) hypostomal carina not lamellate nor with triangular projections (modified in *Genaemirum* Heinrich, 1936, *Leptophatnus* Cameron, 1906, *Oriphatnus* Heinrich, 1967); (4) lower tooth of the mandible lying in the same plane as the upper tooth (bent inward in *Apatetorides* Heinrich, 1938); (5) T2 with punctures (almost completely smooth and impunctate in *Apatetor* Saussure, 1892); (6) area between gastrocoeli bigger than the width of a gastrocoelus (gastrocoeli extremely enlarged in *Stenapatetor* Heinrich, 1938); (7) metascutellum not carinated or carinated only at the base; (8) mandible wide and robust (slender in *Pseudocoelichneumon* Heinrich, 1967); (9) upper tooth longer than lower tooth (shortened in *Liojoppa* Szépligeti, 1908); (10) mesoscutum longer than wide (as long as wide in *Liojoppa* Szépligeti, 1908); (11) metascutellum not globular (globular in *Coeloleptops* Heinrich, 1967); (12) first flagellar segment longer than second (shorter in *Punctileptops* Heinrich, 1967); and (13) area dentipara not well defined (bordered by carinae in *Punctileptops* Heinrich, 1967).

#### ﻿ Protoleptops angolae

Taxon classificationAnimaliaHymenopteraIchneumonidae

(Heinrich, 1967)

D4204041-9E20-53F1-A2CD-59974A9E2EA5

Figs 1A–F , 3A–C

Apatetorops angolae Heinrich, 1967: 83–84 (original description, key); Schmidt and Schmidt 2011: 64 (type catalogue).
Protoleptops angolae ; Townes and Townes 1973: 226 (catalogue, new combination); Yu and Horstmann 1997: 530 (catalogue); Yu et al. 2016 (catalogue).

##### Differential diagnosis.

*Protoleptopsangolae* can be easily distinguished from all the other known species of the genus by the following combination of characters: (1) incomplete carination of propodeum, with costulae lacking and area dentipara confluent with area externa and area spiracularis (carination almost complete in *P.heinrichi*); (2) temples straight and converging (bulging and not converging in *P.farquharsoni*, *P.heinrichi* and *P.magnificus*); (3) white hind tarsus (infuscate in *P.farquharsoni*, *P.heinrichi*, and *P.magnificus*); (4) presence of a small scopa (absent in *P.heinrichi*, bigger in *P.magnificus* and *P.nyeupe* sp. nov.); (5) mesoscutellum reddish-orange (entirely white in *P.nyeupe* sp. nov. and with white lateral marks in *P.magnificus*); (6) T2 medially densely punctate (longitudinally striate in *P.magnificus* and *P.nyeupe* sp. nov.); (7) area petiolaris not delimited (clearly separated from area superomedia in *P.nyeupe* sp. nov.); and (8) mesopleuron densely and strongly punctate (superficially and sparsely punctate in *P.nyeupe* sp. nov.).

**Figure 1.**
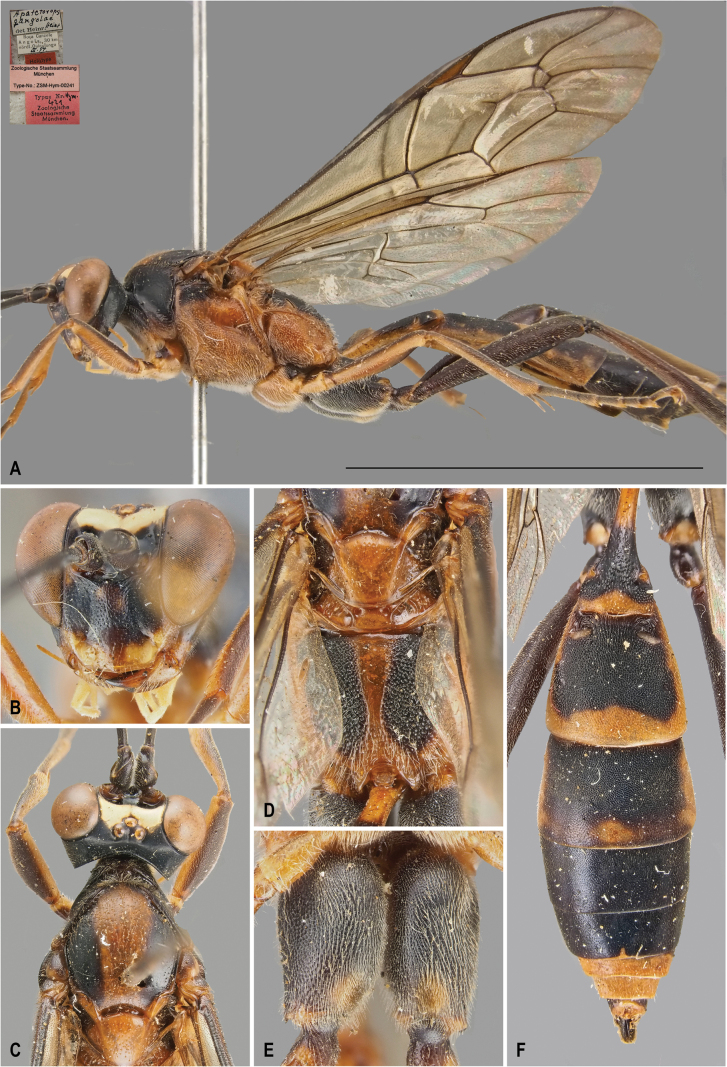
*Protoleptopsangolae* (Heinrich, 1967), female, holotype **A** habitus, lateral view **B** face, frontal view **C** head and mesoscutum, dorsal view **D** mesoscutellum and propodeum, dorsal view **E** hind coxa, ventral view **F** metasomal tergites, dorsal view. Scale bar: 1 cm.

##### Original type series.

***Holotype*** (by original designation). Angola • ♀; Cuanza Norte, Roca Canzele, 30 km north of Quiculungo, Mar. 1954; (ZSM). ***Paratypes*** Angola • 1♀ & 1♂; Cuanza Norte, Roca Canzele, 30 km north of Quiculungo, Mar. 1954; (ZSM).

##### Material examined.

***Holotype*.** Angola • ♀; “[White label] Roca Canzele / Angola, 30 km / nordl.Quiculungo / III. 54 // [White label] *Apatetorops* / ♀ *angolae* / det Heinr. *Heinr* // [Red label] Holotype // [Pink label] Zoologische Staatssammlung / München / Type-No.: ZSM-Hym-00241 // [Red label] Typus Nr. *Hym.* / *421* / Zoologische / Staatsammlung / München.”; (ZSM). ***Paratype*.** Angola • 1♂; “[White label] Roca Canzele / Angola, 30 km / nordl.Quiculungo / III. 54 // [White label] *Apatetorops* / ♂ *angolae* / det Heinr. *Heinr* // [Red label] Allotype; (ZSM).

##### Non-type specimens.

Uganda • 1♀; Kibale N. P., Kanyawara Bio. Station, 00°33'54.4"N, 30°21'29.8"E, 11–18 Apr. 2010, 1509 m, Malaise trap, S. Katusabe & Co. leg. (DDPC).

##### Male.

Described in the original description by Heinrich (1967) as “Allotype”.

##### Distribution.

Angola: Cuanza Norte Province (Heinrich 1967); Uganda: Western Region (new record) (Fig. 8).

##### Remarks.

*Protoleptopsangolae* is hereby recorded for the first time in Uganda, expanding the range of the species from southern Africa to East Africa.

#### ﻿ Protoleptops farquharsoni

Taxon classificationAnimaliaHymenopteraIchneumonidae

(Heinrich, 1967)

4F27A525-82F8-5512-8B40-72F28716B957

Figs 2A–E , 3D–F

Apatetorops farquharsoni Heinrich, 1967: 84–85 (original description, key); Schmidt and Schmidt 2011: 74 (type catalogue).
Protoleptops farquharsoni ; Townes and Townes 1973: 226 (catalogue, new combination); Yu and Horstmann 1997: 530 (catalogue); Yu et al. 2016 (catalogue).

##### Diagnosis of female.

The diagnosis of the female is provided here for the first time based on two females from South Africa (see below in Material examined). Compared to the male, the female has less white patterning overall. The prosternum, mesosternum, and femora are predominantly reddish-brown with only a few scattered yellow patches. In males, these body parts are mostly whitish-yellow. The face is primarily white, with a darker, infuscate area in the center. The orange of the mesoscutum is slightly reduced and the infuscation is more extensive. The posterior yellow band on T2 is smaller and the hind coxa is entirely reddish-brown without any white markings.

**Figure 2.**
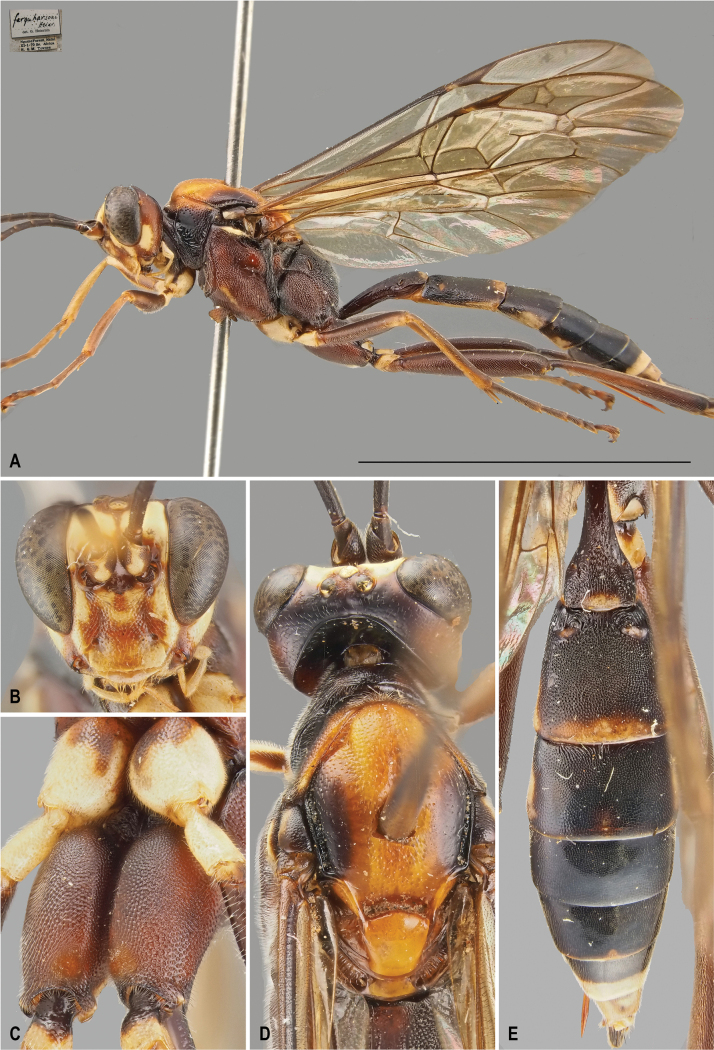
*Protoleptopsfarquharsoni* (Heinrich, 1967), female **A** habitus, lateral view **B** face, frontal view **C** hind coxa, ventral view **D** head, mesoscutum and mesoscutellum, dorsal view **E** metasomal tergites, dorsal view. Scale bar: 1 cm.

##### Differential diagnosis.

*Protoleptopsfarquharsoni* can be easily distinguished from all the other known species of the genus by the following combination of characters: (1) incomplete carination of propodeum, with costulae lacking and area dentipara confluent with area externa and area spiracularis (carination almost complete in *P.heinrichi*); (2) temple, in dorsal view, bulging (straight and converging in *P.angolae* and *P.nyeupe* sp. nov.); (3) hind tarsus brownish-black (white in *P.angolae* and *P.nyeupe* sp. nov.); (4) presence of a small scopa (absent in *P.heinrichi*, bigger in *P.magnificus* and *P.nyeupe* sp. nov.); (5) mesoscutellum reddish-orange (entirely white in *P.nyeupe* sp. nov. and with white lateral marks in *P.heinrichi* and *P.magnificus*); (6) T2 medially densely punctate (longitudinally striate in *P.magnificus* and *P.nyeupe* sp. nov.); and (7) mesoscutum densely punctate anteriorly (sparsely and superficially punctate in *P.magnificus*).

**Figure 3.**
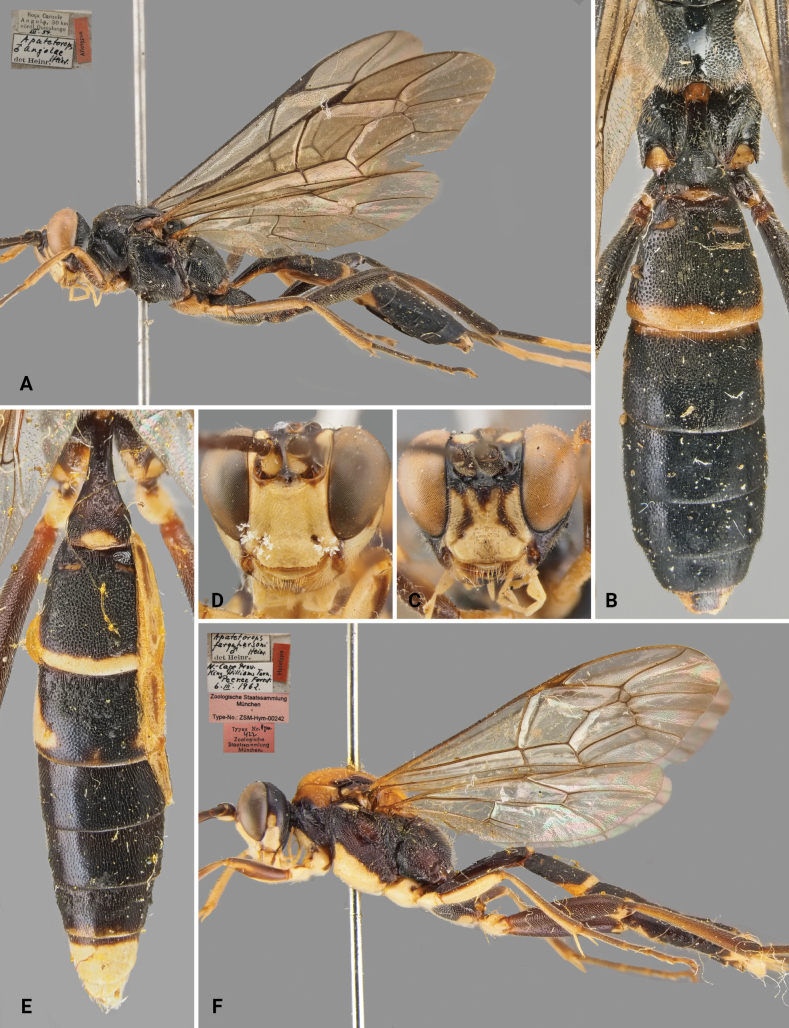
*Protoleptopsangolae* (Heinrich, 1967), male, paratype **A** habitus, lateral view **B** propodeum and metasomal tergites, dorsal view **C** face, frontal view. *Protoleptopsfarquharsoni* (Heinrich, 1967), male, holotype **D** face, frontal view **E** metasomal tergites, dorsal view **F** habitus, lateral view. Scale bar: 1 cm.

##### Original type series.

***Holotype*** (by original designation). South Africa • ♂; Eastern Cape, King William’s Town [now Qonce], Peeree forest, 6 Mar. 1962; (ZSM).

##### Material examined.

***Holotype*.** South Africa • ♂; “[White label] *N.-Cape Prov.* / *King Williams Town* / *Peeree Forest* / *6.III. 1962* // [White label] *Apatetorops* / *farquharsoni* / ♂ *Heinr.* / det Heinr. // [Red label] Holotype // [Pink label] Zoologische Staatssammlung / München / Type-No.: ZSM-Hym-00242 // [Red label] Typus Nr. *Hym.* / *422* / Zoologische / Staatsammlung / München.”; (ZSM).

##### Non-type specimens.

South Africa • 2♀♀; KwaZulu-Natal, Ngome Forest, 1 Nov. 1970, H. & M. Townes leg.; (ZSM).

##### Distribution.

South Africa: Eastern Cape (Heinrich 1967); KwaZulu-Natal (new record) (Fig. 8).

##### Remarks.

The two specimens used for the first female diagnosis of the species had been identified as *Apatetoropsfarquharsoni* by Gerd Heinrich, but the records were never published, despite being integrated into the ZSM collection.

In the original identification key, Heinrich (1967: 81) mentioned “mesosternum uniformly white” as a trait to differentiate *farquharsoni* from *angolae*. However, in the female of the species, the mesosternum is reddish-brown and therefore, the white coloration of the mesosternum should be considered a male-specific trait.

#### ﻿ Protoleptops heinrichi

Taxon classificationAnimaliaHymenopteraIchneumonidae

Heinrich, 1967

C0977A2E-5C31-5CE8-997D-CC39E5047B5F

Figs 4A–E , 6A–C

Protoleptops heinrichi Heinrich, 1967: 72–73 (original description, key, figures); Townes and Townes 1973: 226 (catalogue); Yu and Horstmann 1997: 530 (catalogue); Yu et al. 2016 (catalogue).

##### Differential diagnosis.

*Protoleptopsheinrichi* can be easily distinguished from all the other known species of the genus by the following combination of characters: (1) almost complete carination of the propodeum (incomplete in all the other species); (2) absence of a scopa (present in all the other species); and (3) T2–T5 anteriorly constricted (not constricted in all the other species).

**Figure 4.**
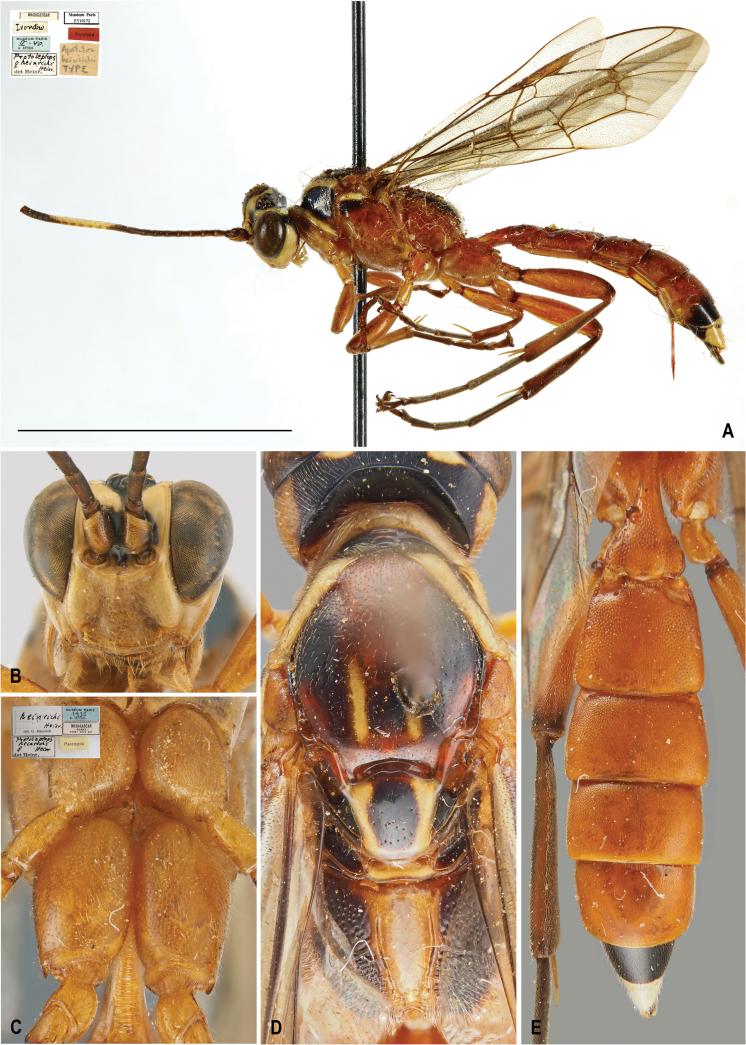
*Protoleptopsheinrichi* Heinrich, 1967, female, holotype **A** habitus, lateral view; female, paratype **B** face, frontal view **C** hind coxa, ventral view **D** head, mesoscutum, mesoscutellum, and propodeum, dorsal view **E** metasomal tergites, dorsal view. Images of the habitus downloaded from the public MNHN database (available at https://science.mnhn.fr/institution/mnhn/collection/ey/item/ey10172). Scale bar: 1 cm.

##### Original type series.

***Holotype*** (by original designation). Madagascar • ♀; Antsiranana, Ivondro, Feb. 1940; (MNHN). ***Paratypes*.** Madagascar • 1♂; Antsiranana, Ivondro, Dec. 1938; MNHN • 1♀; Antsiranana, Rogez, 1935; (ZSM).

##### Material examined.

***Holotype*.** Madagascar • ♀; “[White label] MADAGASCAR // [White label] *Ivondro* // [Blue label] MUSÉUM PARIS / *II.40* / A. SEYRIG // [White label] *Apatetor* / *heinrichi*/ TYPE // [White label] *Protoleptops* / ♀ *heinrichi* / *Heinr.* / det Heinr. // [Red label] Holotype // [Whitel label] Muséum Paris / EY10172”; (MNHN). ***Paratypes*.** Madagascar • ♂; “[White label] MADAGASCAR // [White label] *Ivondro* [Written over Rogez, Foret Cote Est] // [Blue label] MUSÉUM PARIS / *XII.38* / A. SEYRIG // [White label] *Protoleptops* / *heinrichi* ♂ / *Heinr.* / det Heinr. // [Red label] Allotypus // [Whitel label] Muséum Paris / EY10173”; (MNHN) • ♀; “[White label] MADAGASCAR / Rogez / Foret Core Est // [Blue label] MUSÉUM PARIS / *1935* / A. SEYRIG // [White label] *heinrichi* / *Heinr.* / det. G. Heinr. // [White label] *Protoleptops* / *heinrichi* / ♀ *Heinr.* / det Heinr. // [Yellow label] Paratypus”; (ZSM).

##### Male.

Described in the original description by Heinrich (1967) as “Allotype”.

##### Distribution.

Madagascar: Antsiranana (Heinrich 1967) (Fig. 8).

##### Remarks.

The specific epithet given by Heinrich might appear to be self-glorification, as it is named after himself. However, it is actually a dedication to his friend, A. Seyrig, who was the first to recognize it as a new species and labeled it as “*Apatetorheinrichi*”, but never officially described it. When Heinrich discovered the species at the MNHN, he chose to retain the name in honor of his friend’s intentions, stating: “I felt bound to carry out the will of my late friend, rather than to shrink from the possibility of being blamed for self-glorification in using this species name” (Heinrich 1967: 72).

#### ﻿ Protoleptops magnificus

Taxon classificationAnimaliaHymenopteraIchneumonidae

(Heinrich, 1967)

1C614B6A-4596-5F35-9F12-9C69A833A4EC

Figs 5A–F , 6D–F

Apatetorops magnificus Heinrich, 1967: 81–83 (original description, key, figures); Schmidt and Schmidt 2011: 83 (type catalogue).
Protoleptops magnifica [sic]; Townes and Townes 1973: 226 (catalogue, new combination, incorrect gender agreement).
Protoleptops magnificus ; Yu and Horstmann 1997: 530 (catalogue, mandatory change); Yu et al. 2016 (catalogue).

##### Differential diagnosis.

*Protoleptopsmagnificus* can be easily distinguished from all the other known species of the genus by the following combination of characters: (1) incomplete carination of the propodeum, with costulae lacking and area dentipara confluent with area externa and area spiracularis (carination almost complete in *P.heinrichi*); (2) temple, in dorsal view, bulging (straight and converging in in *P.angolae* and *P.nyeupe* sp. nov.); (3) hind tarsus infuscate (white in *P.angolae* and *P.nyeupe* sp. nov.); (4) presence of a scopa taking up 2/3 of the ventral part of the coxa (absent in *P.heinrichi*, reduced in *P.angolae* and *P.farquharsoni*); (5) mesoscutellum with lateral white marks (reddish-orange in *P.farquharsoni* and *P.angolae*); (6) T2 longitudinally striate medially (densely punctate in *P.angolae* and *P.farquharsoni*); and (7) mesoscutum sparsely and superficially punctate (densely punctate anteriorly in *P.farquharsoni*).

**Figure 5.**
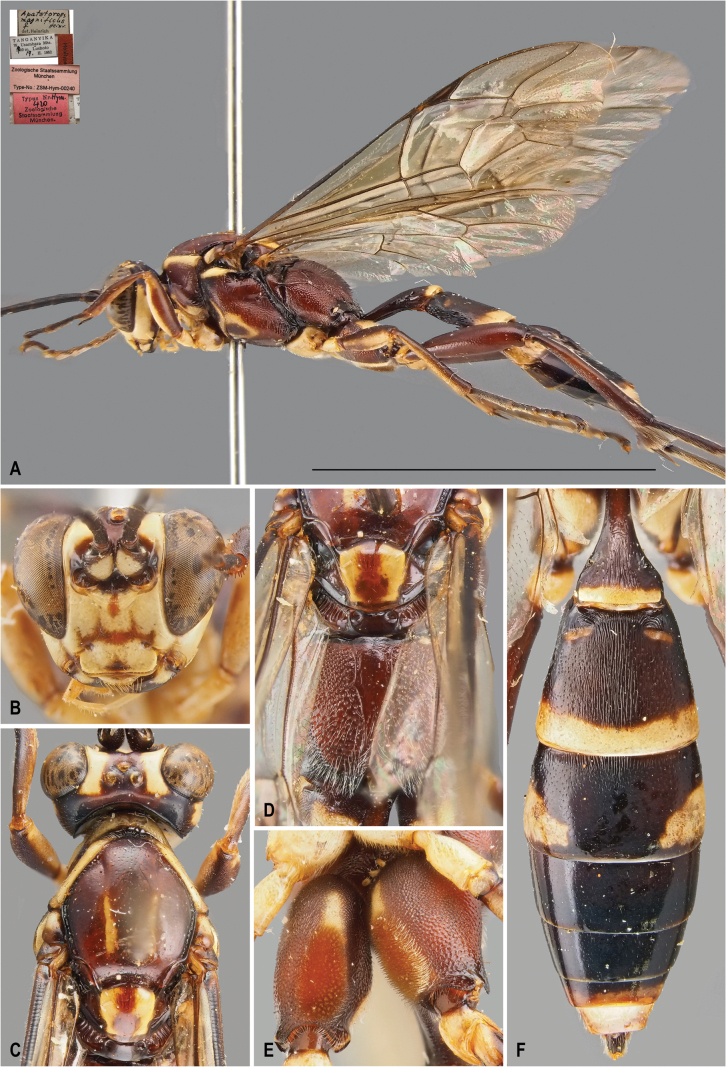
*Protoleptopsmagnificus* (Heinrich, 1967), female, holotype **A** habitus, lateral view **B** face, frontal view **C** head, mesoscutum and mesoscutellum, dorsal view **D** propodeum, dorsal view **E** hind coxa, ventral view **F** metasomal tergites, dorsal view. Scale bar: 1 cm.

##### Original type series.

***Holotype*** (by original designation). Tanzania • ♀; Tanga, West Usambara Mountains, Lushoto, 1700 m 19 Feb. 1962; (ZSM).

**Figure 6.**
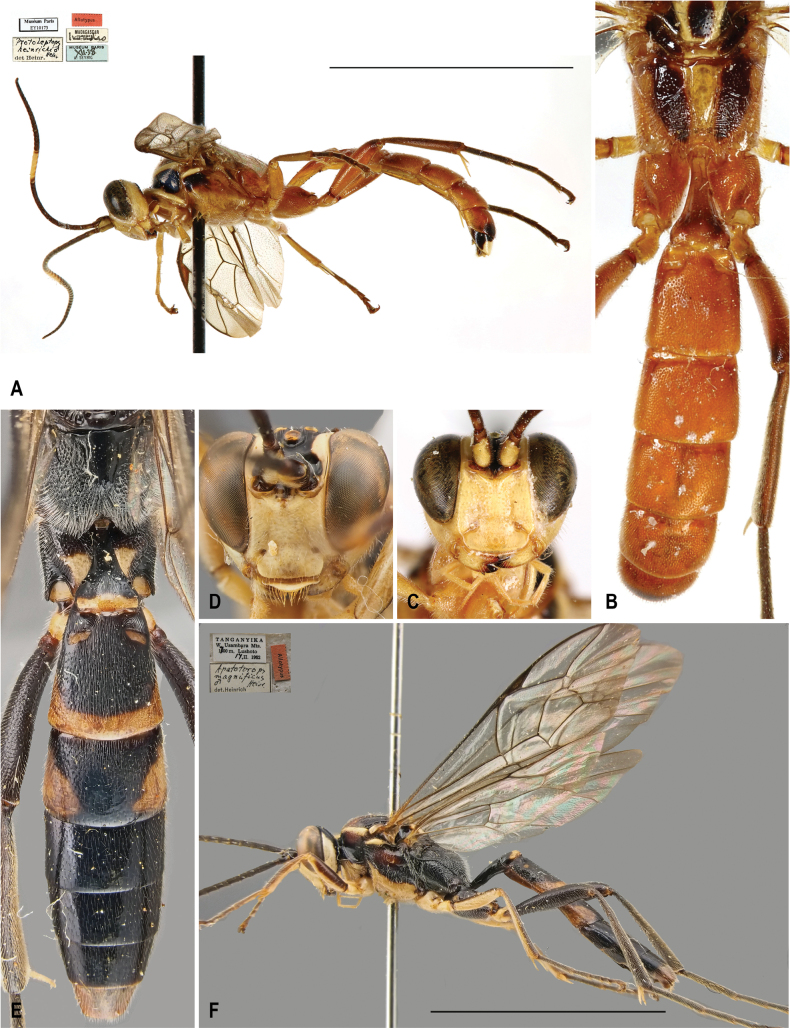
*Protoleptopsheinrichi* Heinrich, 1967, male, paratype **A** habitus, lateral view **B** propodeum and metasomal tergites, dorsal view **C** face, frontal view. Downloaded from the public MNHN database (available at https://science.mnhn.fr/institution/mnhn/collection/ey/item/ey10173). *Protoleptopsmagnificus* (Heinrich, 1967), male, paratype, **D** face, frontal view **E** propodeum and metasomal tergites, dorsal view **F** habitus, lateral view. Scale bar: 1 cm.

***Paratypes*.** Tanzania • 5♀♀ & 1♂; same locality as the holotype, 17 Feb. 1962; ZSM • 1♀; same locality as the holotype, 1600 m, 5 Mar. 1962; (ZSM). South Africa • 1♀; Eastern Cape, Port St. Johns, 20 Feb. 1963; (ZSM)

##### Material examined.

***Holotype*.** Tanzania • ♀; “[White label] TANGANYIKA / W Usambara Mts. / 1700 m. Lushoto / *19*.II.1962 [White label] *Apatetorops* / *magnificus* / ♀ *Heinr.* / det.Heinr. // [Red label] Holotype // [Pink label] Zoologische Staatssammlung / München / Type-No.: ZSM-Hym-00240 // [Red label] Typus Nr. *Hym.* / *420* / Zoologische / Staatsammlung / München.”; (ZSM). ***Paratypes*.** Tanzania • ♂; “[White label] TANGANYIKA / W Usambara Mts. / 1700 m. Lushoto / *17*.II.1962 [White label] *Apatetorops* / *magnificus* / ♂ *Heinr.* / det.Heinr. // [Red label] Allotypus”; (ZSM).

##### Non-type specimens.

South Africa • 1♀; Mpumalanga, Waterval-Boven [=Emgwenya], Elandsrivier, 18.i.2000, J. Halada leg.; (OÖLM).

##### Male.

Described in the original description by Heinrich (1967) as “Allotype”.

##### Distribution.

South Africa: Eastern Cape (Heinrich 1967); Mpumalanga (new record); Tanzania: Tanga region (Heinrich 1967) (Fig. 8).

##### Remarks.

In their catalogue, Townes and Townes (1973: 226) provided a new combination for *Apatetoropsmagnificus* Heinrich, 1967, moving the species into the genus *Protoleptops*. While doing so, they referred to *Protoleptopsmagnifica* [sic], possibly misinterpreting the gender of the genus *Protoleptops* as feminine instead of masculine. Subsequently, Yu and Horstmann (1997: 530) correctly interpreted the gender as masculine and made the mandatory change to the suffix in accordance with ICZN (1999: article 34.2).

#### ﻿ Protoleptops nyeupe

Taxon classificationAnimaliaHymenopteraIchneumonidae

Dal Pos & Di Giovanni sp. nov.

D55DA95B-6FE4-5D54-BFDB-C967E167D4BF

https://zoobank.org/54F974F4-597C-46A2-B817-0EB76421D494

Fig. 7A–E

##### Type material.

***Holotype*** • ♀, “[White label] BURUNDI. Rwegura, Kibira / Nat. Park, 2 53 25.9S 29 27 25.4E, / 2226 m, 28-30.I.2011, M. Mei, / P. Cerretti, D. Withmore [Whitmore] leg. // [Red label] HOLOTYPE / *Protoleptops* / *nyeupe* / Dal Pos & Di Giovanni, des. 2024 // FSCA 00051872” (FSCA). The specimen is in perfect condition. ***Paratype*** • ♀, same data as the holotype. The specimen is in perfect condition (MZUR).

**Figure 7.**
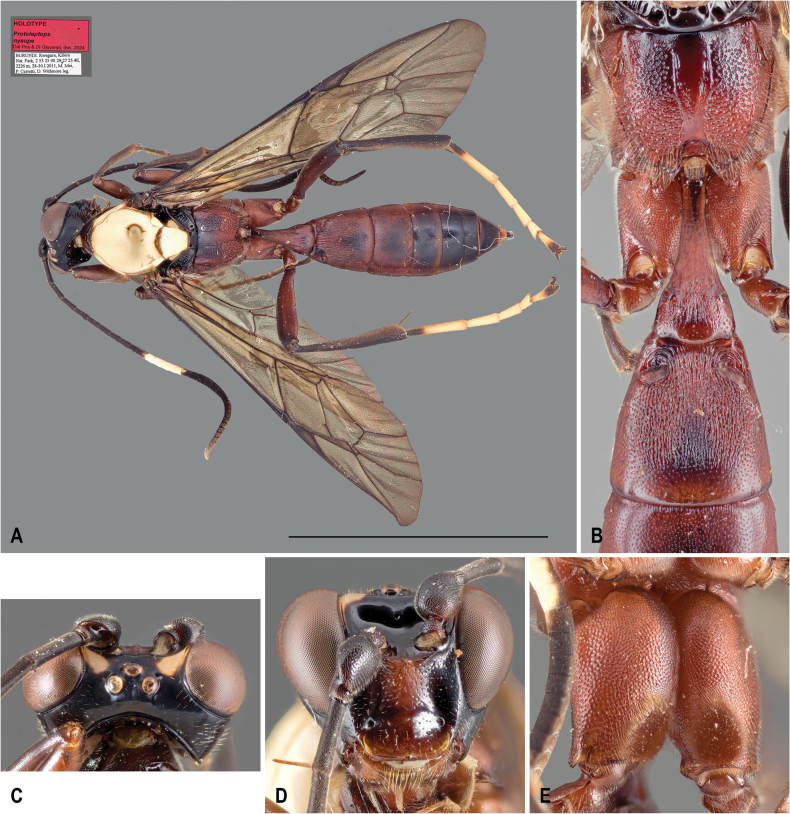
*Protoleptopsnyeupe* Dal Pos & Di Giovanni, sp. nov., female, holotype **A** habitus, dorsal view **B** propodeum and T1-T2, dorsal view **C** head, dorsal view **D** face, frontal view **E** hind coxa, ventral view. Scale bar: 1 cm.

##### Differential diagnosis.

*Protoleptopsnyeupe* sp. nov. can be easily distinguished from all the other known species of the genus by the following combination of characters: (1) incomplete carination of the propodeum, with costulae lacking and area dentipara confluent with area externa and area spiracularis (almost complete in *P.heinrichi*); (2) temples straight and converging (bulging and not converging in *P.farquharsoni*, *P.heinrichi* and *P.magnificus*); (3) white hind tarsus (infuscate in *P.farquharsoni*, *P.heinrichi*, and *P.magnificus*); (4) presence of a scopa taking up 1/3 of the ventral part of the coxa (absent in *P.heinrichi*, reduced in *P.angolae* and *P.farquharsoni*, and taking up 2/3 of the ventral side of the coxa in *P.magnificus*); (5) mesocutellum entirely white (reddish-orange in *P.farquharsoni* and *P.angolae*; with lateral white marks in *P.heinrichi* and *P.magnificus*); (6) T2 medially longitudinally striate (densely punctate in *P.angolae* and *P.farquharsoni*); (7) area petiolaris well delimited (not well delimited in *P.angolae* sp. nov.); and (8) mesopleuron superficially and sparsely punctate (densely and strongly punctate in *P.angolae*).

##### Etymology.

The specific epithet *nyeupe* is a noun in apposition, derived from the Swahili word “nyeupe” for white. This name refers to the extensive white coloration of the mesoscutum and mesoscutellum, which stands in stark contrast to the dark coloration of the rest of the body.

##### Description.

Holotype female. Body length: 17.8 mm; fore wing length: 14.2 mm. ***Head*.** Overall shining; face subquadrate, as wide as medially high, smooth, with very sparse and superficial punctures, medio-apically protruding in a very distinct blunt tubercle right below antennal sockets, clear delimitation between clypeus and face present; frons concave, smooth and shining; vertex matt and impunctate; ocellar triangle equilateral, elevated and proximally delimited by a shallow sulcus; ocular-ocellar distance about 1.3 × ocellus diameter, inter-ocellar distance 1.0 × ocellus diameter; occipital carina distinct and complete, meeting hypostomal carina at base of mandible; temples straight and converging in dorsal view; gena, in lateral view, not strongly inflated, matt; clypeus medially slightly convex in lateral view, shining with straight apical margin and almost completely impunctate; malar space about 0.7 × basal width of mandible; malar sulcus present and shagreened; mandible robust, with sparse setiferous punctures centrally, teeth rather stout and widely separated with ventral tooth shorter (about 0.5 ×) than upper tooth; maxillary palp long, reaching fore coxa, 5^th^ segment about 1.5 × as long as 4^th^; antenna with 45 flagellomeres, slightly enlarged, with flagellomeres 20–38 ventrally flattened and 1.4 × as wide as long, 1^st^ flagellomere about 1.3 × as long as 2^nd^, apical flagellomere distinctly longer than wide. ***Mesosoma***. Overall shining; pronotum with shallow punctures; epomia present and strong; propleuron smooth, with dense, shallow punctures and covered with setae, projected into a blunt, rounded flange ventro-apically; mesoscutum subquadrate, smooth, impunctate, notauli absent; mesoscutellum not elevated over metascutellum, impunctate and not carinated; mesopleuron shining on upper 1/3, with shallow and sparse punctures, more densely and finely punctate ventrally, on upper-posterior section with a deep sulcus right below subtegular ridge; epicnemial carina continuous with subtegular ridge; subtegular ridge strongly projecting outwardly anteriorly; sternaulus absent; posterior transverse carina of mesosternum completely absent; metapleuron with dense, shallow punctures, juxtacoxal carina absent; propodeum short in lateral view, sloping gently with almost no horizontal portion, overall irregularly sculptured throughout except for anterior margin and for area basalis and area petiolaris, which are completely smooth and almost shining; lateral longitudinal carina present throughout length of propodeum; lateromedian longitudinal carina present; anterior transverse carina absent so that area basalis and area superomedia are continuous; posterior transverse carina present only medially, delimiting a small area petiolaris. ***Legs*.** All coxae setose; fore and middle coxae ventrally impunctate; hind coxa with dense punctures throughout; scopa present, occupying 1/3 of apico-ventral region of hind coxa. Hind femur about 5.3 × as long as medially high. Tarsal claws without pecten. ***Wings*.** Fore wing with 3rs-m present, areolet rhomboidal, with 3rs-m and 2rs-m converging; 1cu-a opposite M&RS, CU between 1m-cu&M and 2cu-a about 1.5 × as long as 2cu-a. Hind wing with distal abscissa of CU present, pigmented, CU about 3.5 × as long as cu-a. ***Metasoma*.** T1 shining throughout, with postpetiole longitudinally striate except shagreened posterior portion with sparse punctures; T2 with gastrocoeli deep and subquadrate; thyridia present, space between gastroceoli narrower than one gastrocoelus; T3 superficially and densely punctate, impunctate posteriorly; remaining tergites shagreened; terebra (i.e., external visible portion of the ovipositor) short, with densely setose ovipositor sheaths. ***Coloration*.** Head black with central area of face, clypeus and mandible (except black apical teeth) reddish-brown; two white comma-shaped patches on frons running from frontal orbit towards ocellar triangle. Scape and pedicel black with only a reddish-brown patch; flagellum black with white annulus present only on dorsal side, from 10^th^ to 15^th^ flagellomeres. Mesosoma reddish-brown, with dorso-lateral portion of propleuron, entire mesoscutum, and mesoscutellum white; dorsal portion of mesopleuron, metanotal trough and anterior portion of propodeum infuscate. Legs overall reddish-brown, with dorsal sides of all femora and fore and mid tibiae infuscate; hind tibia, fore and mid tarsi, black; hind tarsus white with only proximal part of basitarsus, distal portion of telotarsus and claws black. Wing entirely hyaline with pterostigma centrally light brown. Metasoma with T1–T3 reddish-brown with only an infuscate patch on postero-median portion of T2 and T3; T4 infuscate; T4–T8 black.

**Male.** Unknown.

##### Host.

Unknown.

##### Distribution.

**Figure 8.**
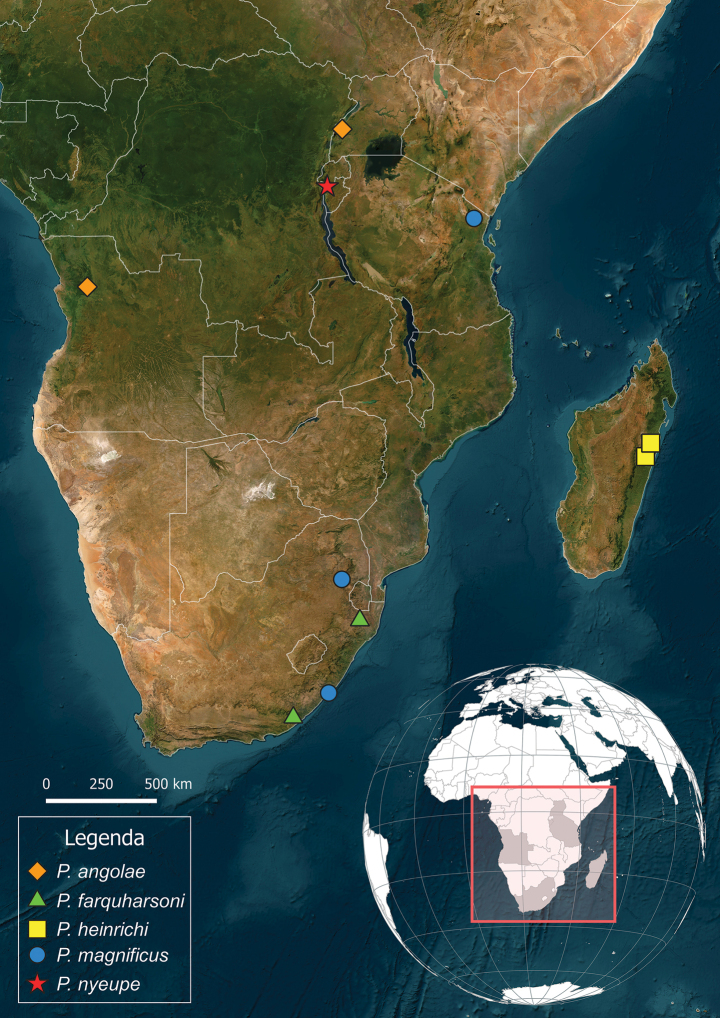
Distribution map of the known species of the genus *Protoleptops* Heinrich, 1967.

Burundi: Cibitoke Province (Fig. 8).

## ﻿Discussion

The discovery of the new species occurred in the bushes at the edge of a dirt road, where specimens of *P.nyeupe* sp. nov. were collected together with some females of an unidentified Cryptini (Ichneumonidae, Cryptinae), with which the new species shares an absolutely identical color pattern (i.e., creamy white mesoscutum in sharp contrast to the dark coloration of the rest of the body). The two species, *P.nyeupe* sp. nov. and the unidentified Cryptinae, markedly stood out from the background as small whitish moving spheres (M. Mei pers. obs.). From our observations in different museums and collections, various species and genera of both Ichneumoninae and Cryptinae share this distinctive color pattern in the Afrotropics. However, the significance of this unusual mimetic chain is likely to remain unanswered until a deeper understanding of the taxonomy and biology of Darwin wasps in the Afrotropics is achieved. Of note, the discovery of *P.nyeupe* sp. nov. also marks the first record of the subfamily Ichneumoninae for Burundi. This “surprising” finding, coupled with the first record of *P.angolae* in East Africa, shows that knowledge about the diversity and distribution of Darwin wasps in the Afrotropical region is still severely lacking. Indeed, beyond a small number of nations that, for historical reasons, have been reasonably sampled, most ecotypes and countries in the Afrotropics have not been adequately investigated yet (Meier et al. 2024).

## Supplementary Material

XML Treatment for Protoleptops

XML Treatment for Protoleptops angolae

XML Treatment for Protoleptops farquharsoni

XML Treatment for Protoleptops heinrichi

XML Treatment for Protoleptops magnificus

XML Treatment for Protoleptops nyeupe

## ﻿Additional information

### Author ORCIDs

Davide Dal Pos https://orcid.org/0000-0002-9122-934X

Augustijn De Ketelaere https://orcid.org/0009-0007-0260-5483

Filippo Di Giovanni https://orcid.org/0000-0002-9811-5599

### Data availability

All of the data that support the findings of this study are available in the main text.
